# *Tigriopus iranicus* sp. nov., a new species of Harpacticidae (Copepoda, Crustacea) from Iran, with a redescription of *T. raki* Bradford, 1967

**DOI:** 10.3897/zookeys.1035.61584

**Published:** 2021-04-27

**Authors:** Fatemeh Nazari, Omid Mirshamsi, Pedro Martínez Arbizu

**Affiliations:** 1 Department of Biology, Faculty of Science, University of Jiroft, Jiroft, Iran University of Jiroft Jiroft Iran; 2 Department of Biology, Faculty of Science, Ferdowsi University of Mashhad, Mashhad, Iran Ferdowsi University of Mashhad Mashhad Iran; 3 German Centre for Marine Biodiversity Research DZMB, Südstrand 44, 26382 Wilhelmshaven, Germany German Centre for Marine Biodiversity Research Wilhelmshaven Germany

**Keywords:** Harpacticoida, meiofauna, Oman Sea, Persian Gulf, rocky shore, splash pool, taxonomy

## Abstract

The first representative of *Tigriopus* Norman, 1869 from the north-western Indian Ocean is described from rock pools on the Iranian coast. *Tigriopus iranicus***sp. nov.** is distinguishable from its congeners by i) the possession of two maxillary endites, each with two setae; ii) a two-segmented mandibular endopod; iii) P1enp-3 with one pinnate claw, a well-developed geniculate spine and a small seta; and iv) female P6 with two setae. Additionally, we present a complete redescription of *Tigriopus raki* Bradford, 1967 on the basis of paratype material and a key to the species of the genus.

## Introduction

**Figure 1.**
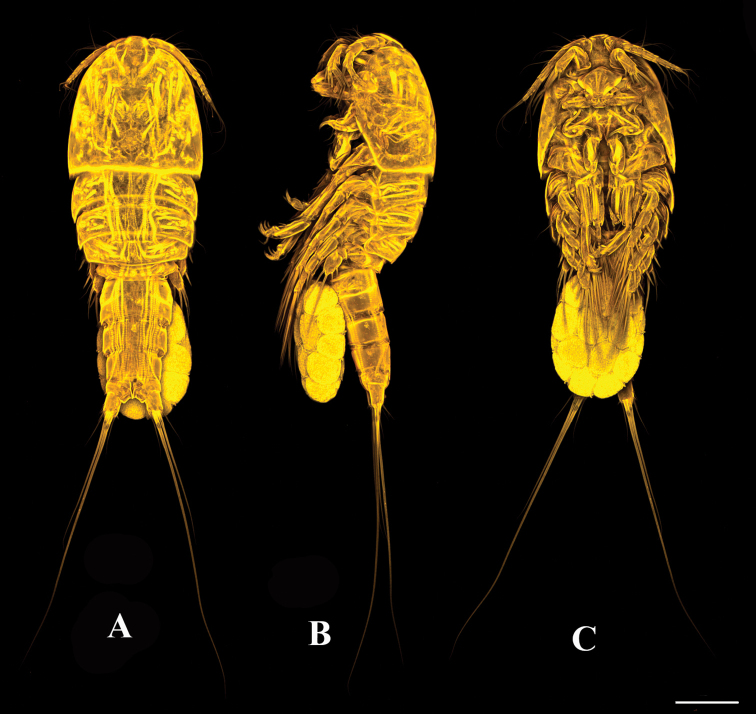
*Tigriopus iranicus* sp. nov. female. Confocal laser microphotograph **A** habitus, dorsal **B** habitus, lateral **C** habitus, ventral. Scale bar: 100 µm.

**Figure 2.**
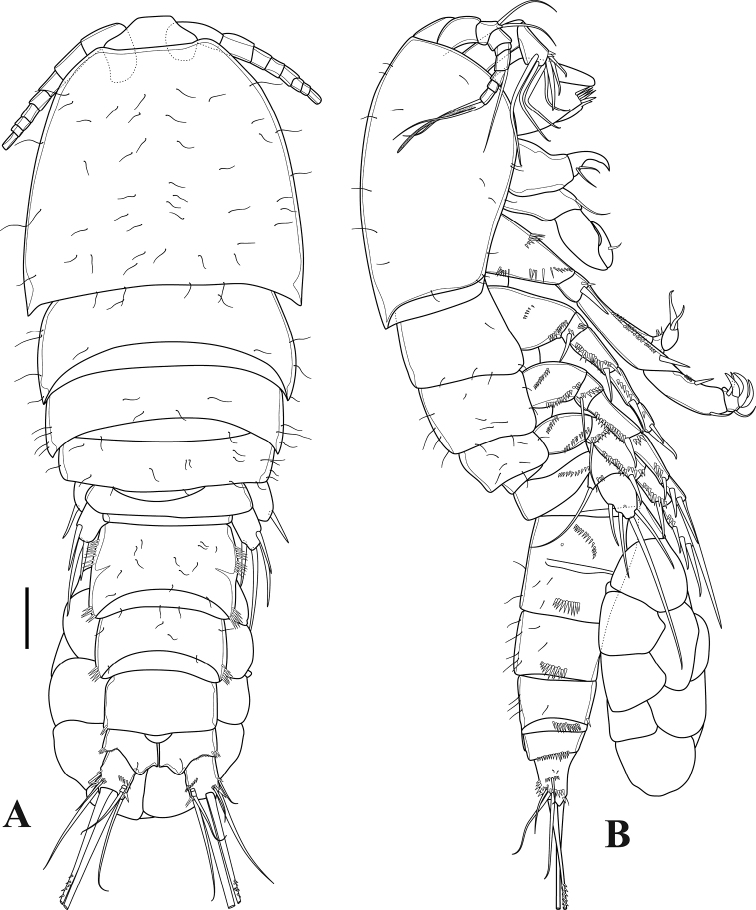
*Tigriopus iranicus* sp. nov. female **A** habitus, dorsal **B** habitus, lateral. Scale bar: 50 µm.

**Figure 3.**
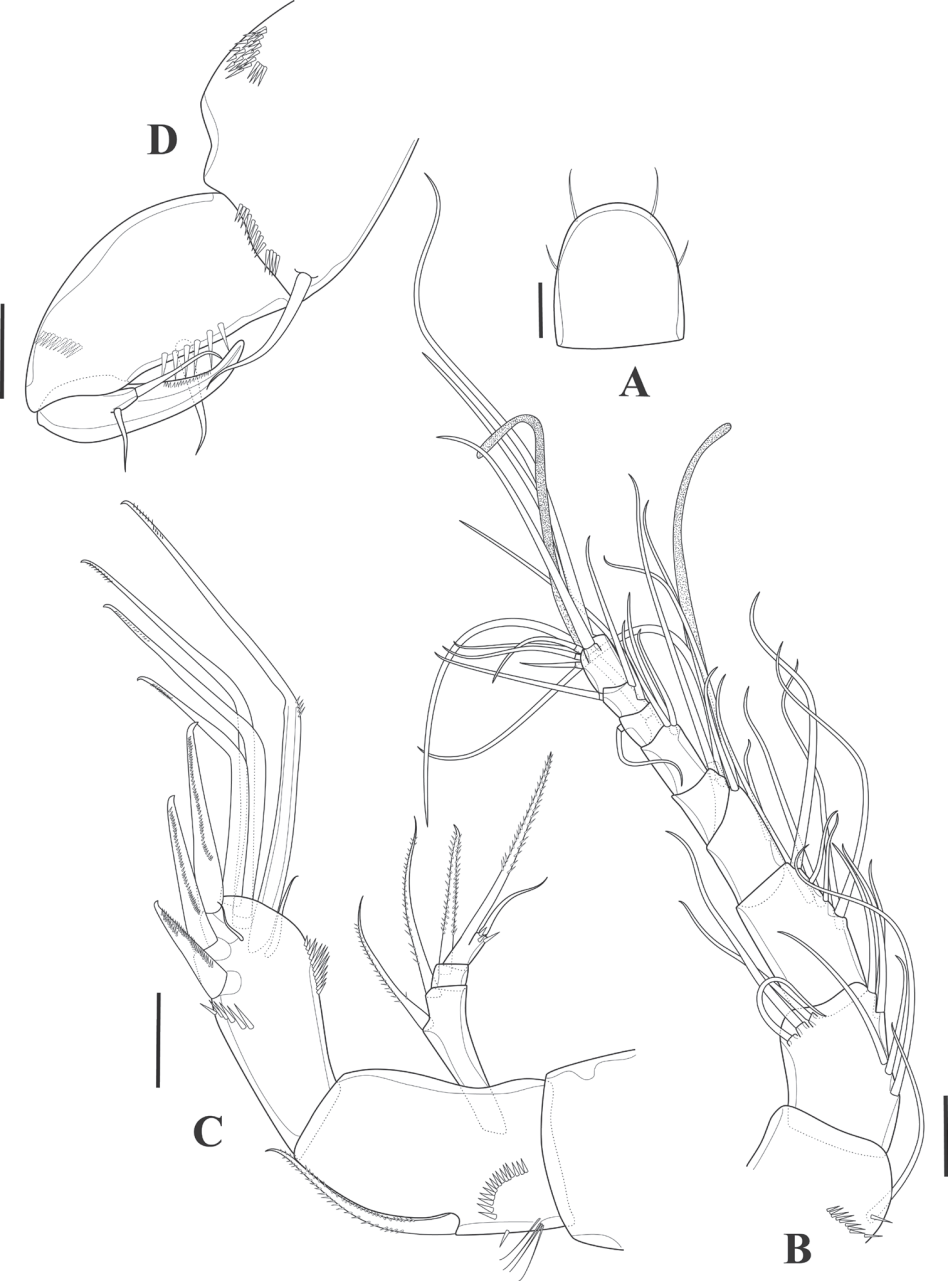
*Tigriopus iranicus* sp. nov. female **A** rostrum, dorsal **B** antennule **C** antenna **D** maxilliped. Scale bars: 20 µm.

**Figure 4.**
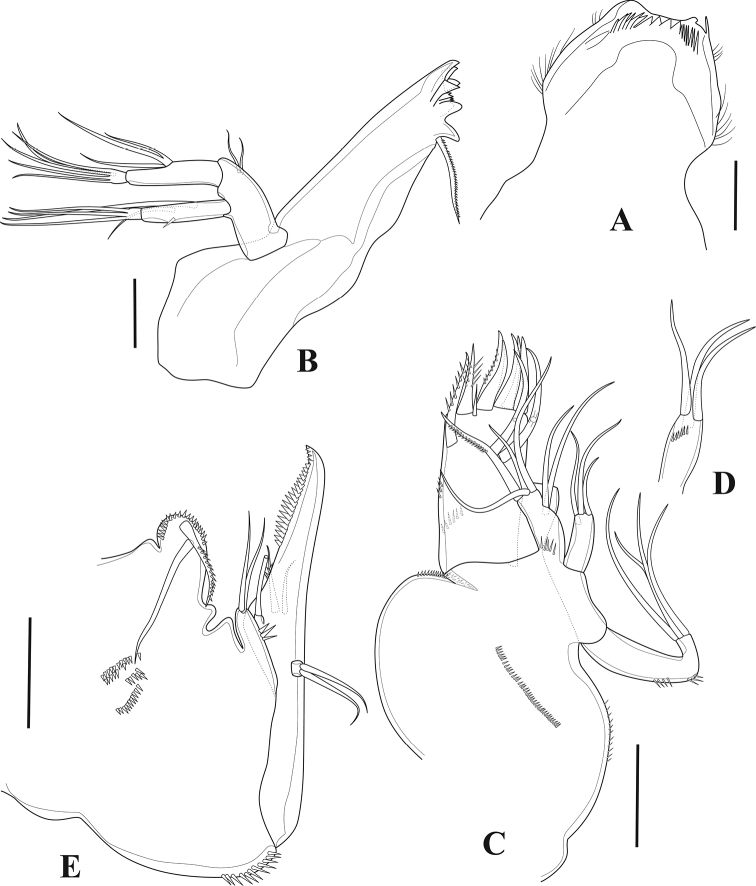
*Tigriopus iranicus* sp. nov. female **A** labrum **B** mandible **C** maxillule **D** coxal endite of the maxillule **E** maxilla. Scale bars: 20 µm.

**Figure 5.**
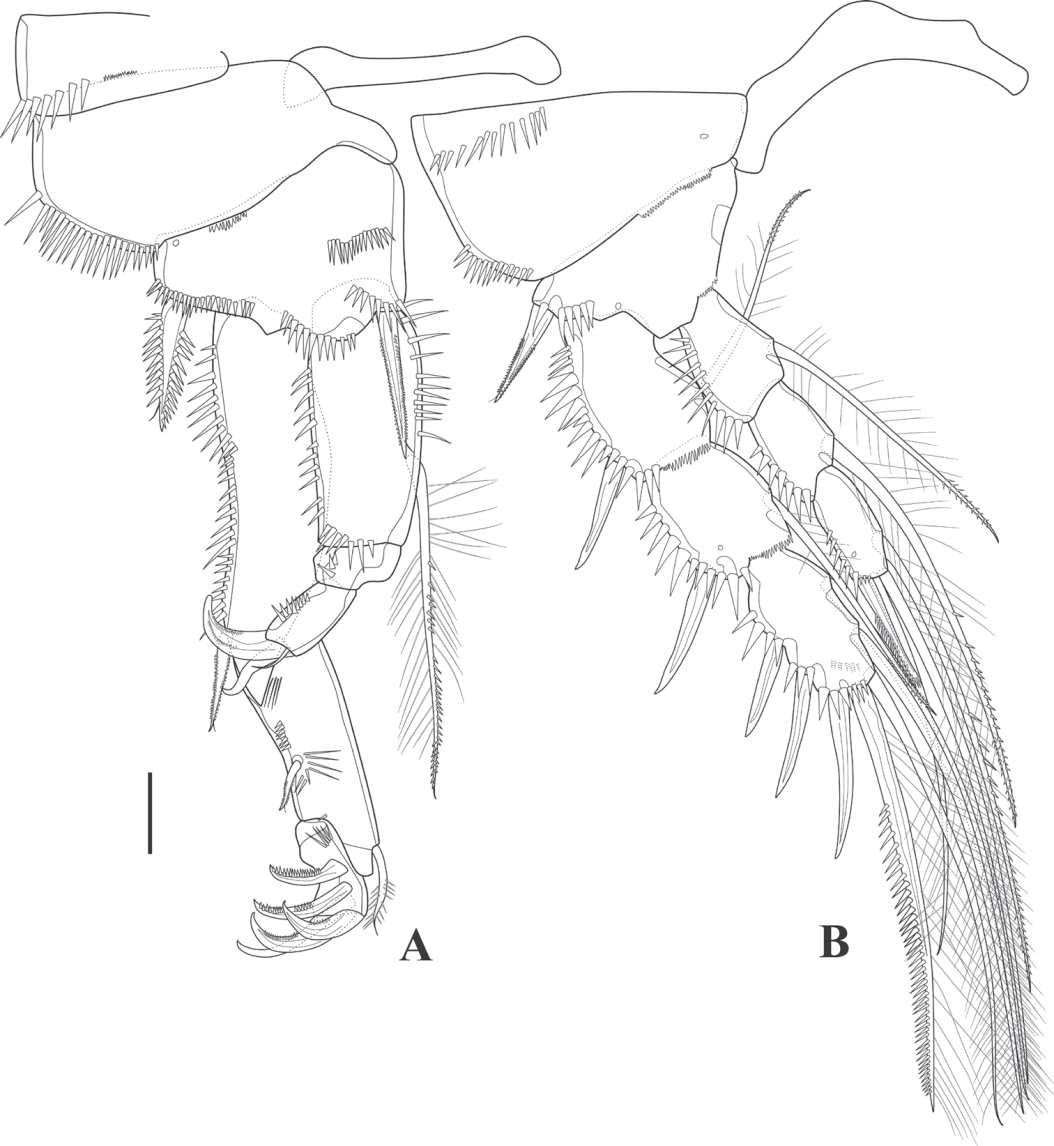
*Tigriopus iranicus* sp. nov. female **A**P1**B**P2. Scale bar: 20 µm.

**Figure 6.**
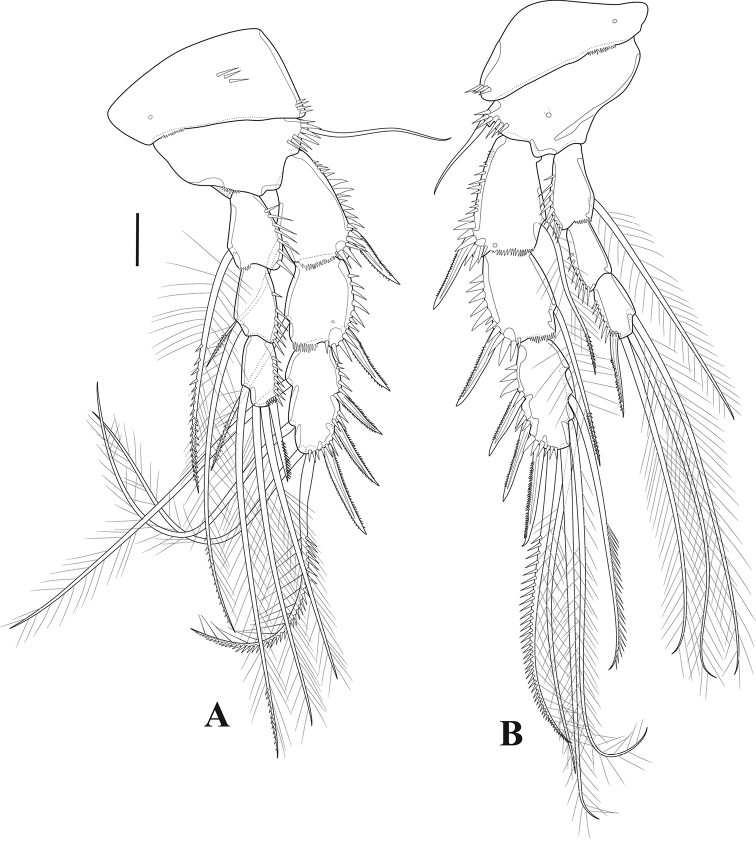
*Tigriopus iranicus* sp. nov. female **A**P3**B**P4. Scale bar: 20 µm.

**Figure 7.**
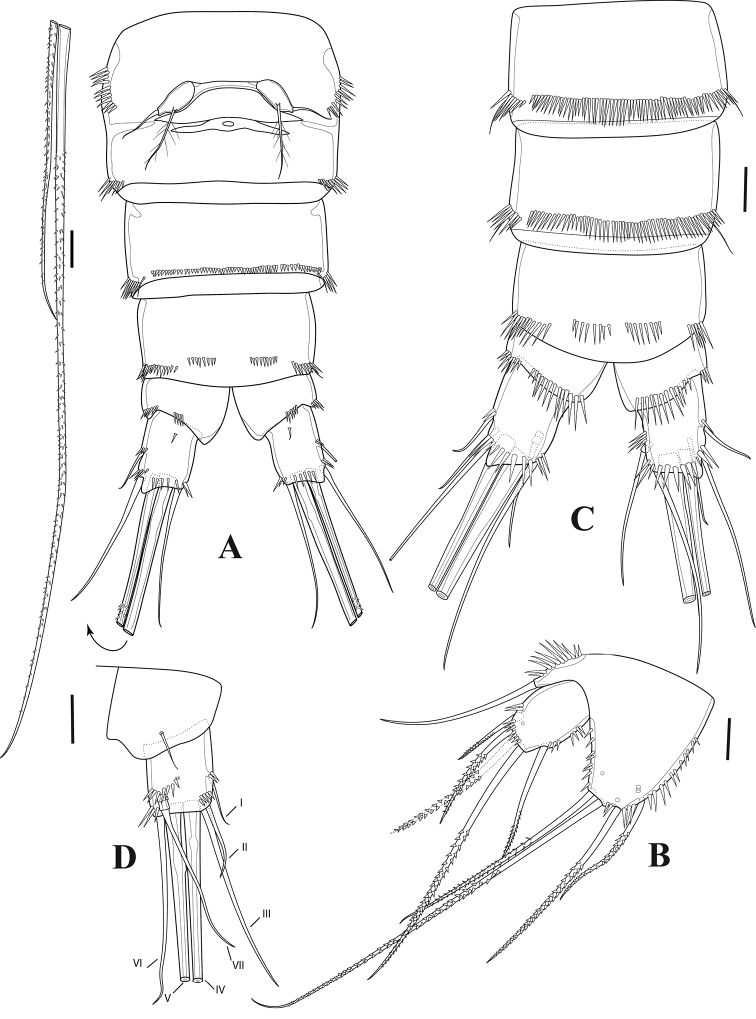
*Tigriopus iranicus* sp. nov. female **A** urosome, ventral **B**P5. Male **C** urosome, ventral **D** furcal ramus, dorsal. Scale bars: 20 µm.

**Figure 8.**
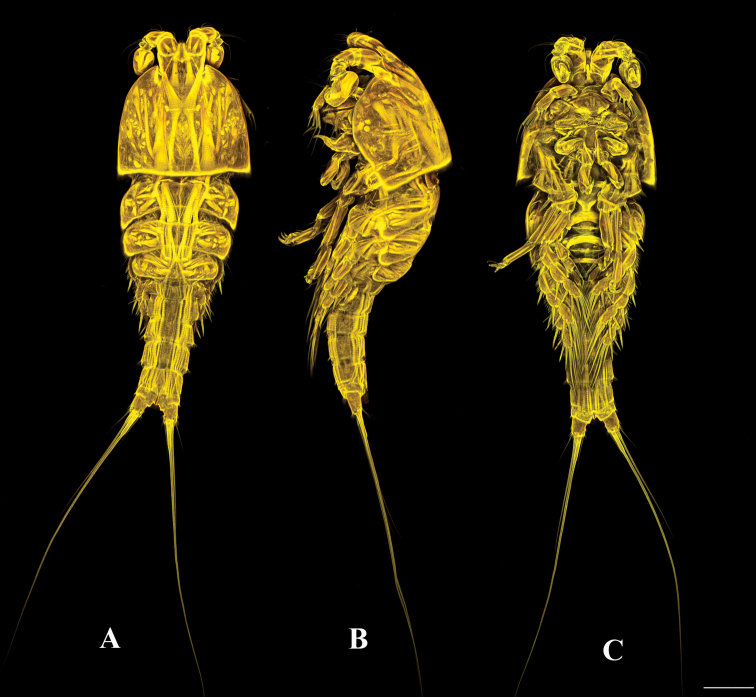
*Tigriopus iranicus* sp. nov. male. Confocal laser microphotograph **A** habitus, dorsal **B** habitus, lateral **C** habitus, ventral. Scale bar: 100 µm.

**Figure 9.**
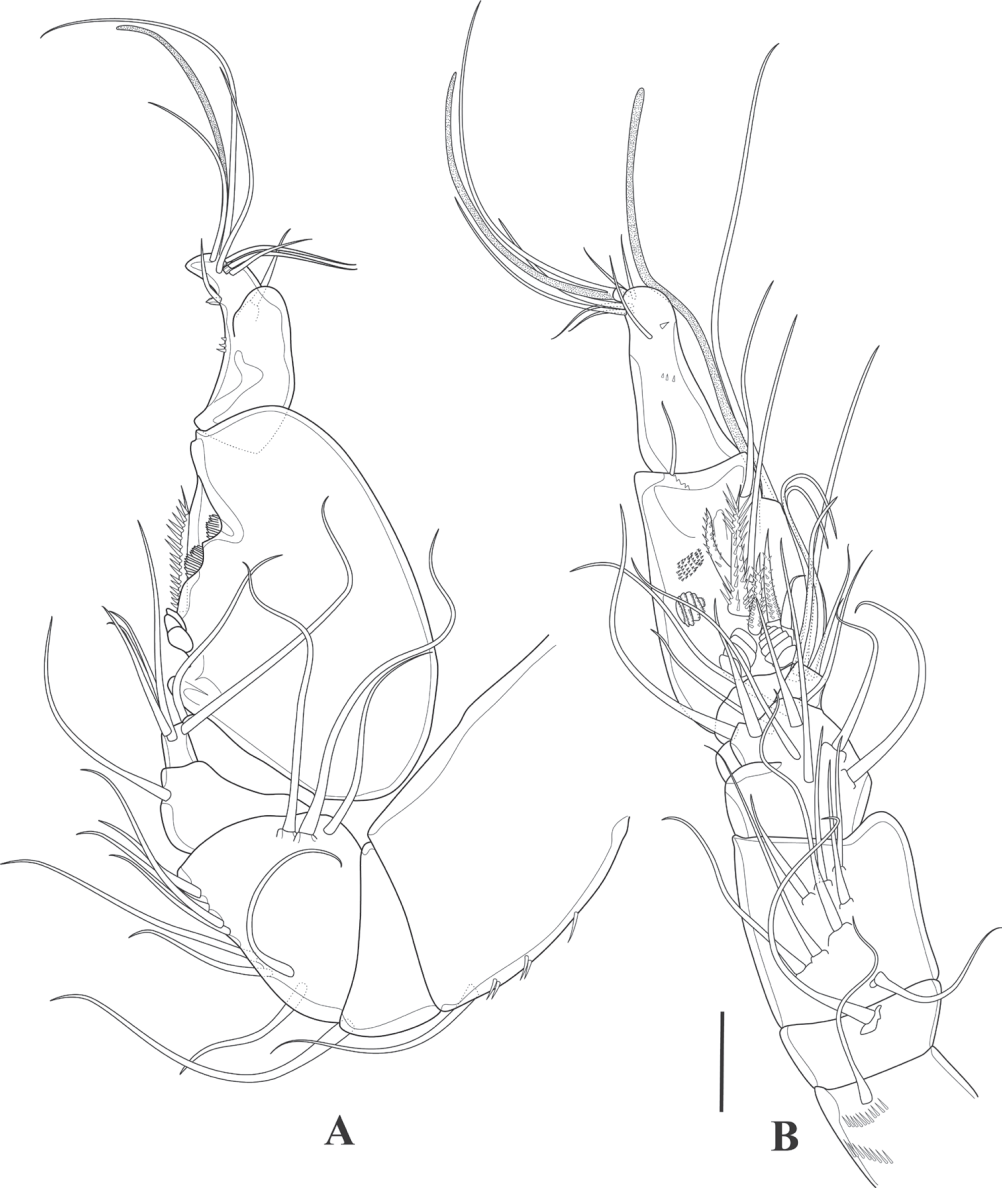
*Tigriopus iranicus* sp. nov. male **A** antennule, ventral **B** antennule, lateral view. Scale bar: 20 µm.

**Figure 10.**
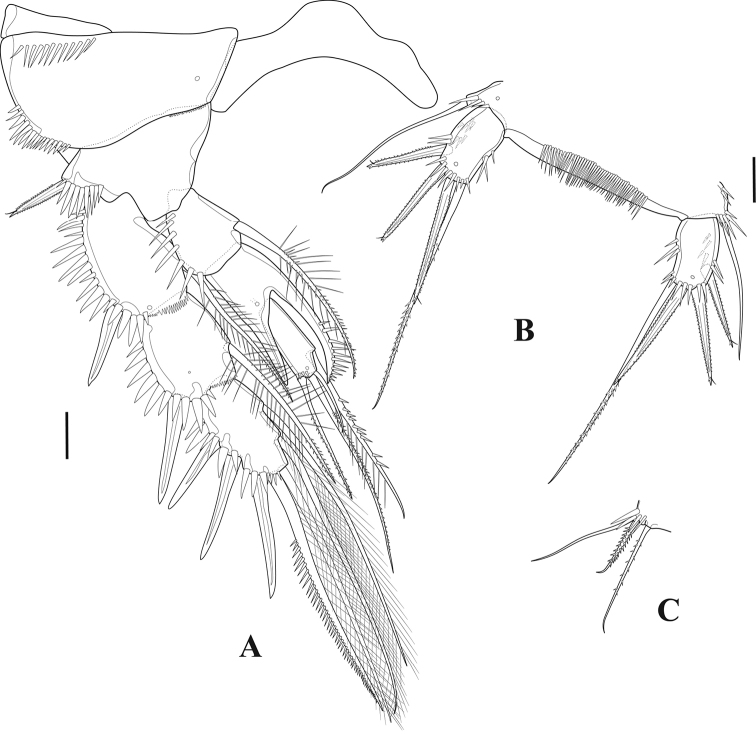
*Tigriopus iranicus* sp. nov. male **A**P2**B**P5**C**P6. Scale bars: 20 µm.

The genus *Tigriopus* was introduced by Norman (1869) based on a specimen of *T. lilljeborgi* Norman, 1869 (currently accepted as *T. fulvus* (Fischer, 1860)) with strong claws on the P1, from Shetland, on the coast of Scotland. *Tigriopus brevicornis* (Müller, 1776) (= *Cyclops brevicornis* Müller, 1776), from the intertidal zone of the Danish coast, is without any doubt one of the oldest described members of the genus. Lang (1948: 311) claimed that the first member of the genus to be described is that illustrated by Ström (1765: 590, pl. IX, figs 1–10) and later described as *Cyclops brevicornis* by Müller (1776). Lang’s claim (1948) is based on Ström’s (1765) illustrations and his explanation of the habitat of the specimen, which was found in rocky pools but not in the open sea. The type species of *Tigriopus*, *Harpacticus fulvus* Fischer, 1860, which was originally described from Madeira, was reallocated to *Thalestris* Claus, 1863 by Claus (1863). Morphological similarities between *T. brevicornis* and *T. fulvus* (e.g. five setae on the female P5 baseoendopod, the male P5 with four setae on the exopod and one seta on the baseoendopod) caused Lang (1948: 340) to synonymize the nominotypical *T. fulvus*, T. fulvus var. adriatica Douwe, 1913 from Rovinj (Croatia) (Douwe 1913), and T. fulvus var. algirica Monard, 1936 from Algeria (Monard 1936) with *T. brevicornis*; only *T. brevicornis* was thought to be present in Europe until 1960. Moreover, a variety of *T. fulvus*, *northumbriensis*, was recorded from Scotland by Mistakidis (1949). Upon the inspection of micromorphological characters, Bozic (1960) recognized two European species: the northern, *T. brevicornis*, and the Mediterranean, *T. fulvus*, and Carli and Fiori (1977) supported the separation of these two species based on few morphological differences. Some years later, Soyer et al. (1987) observed the presence of five groups of species based on their geographic distributions and on some morphological similarities. Recent genetic studies on the Mediterranean population of *T. fulvus* revealed the presence of a single species with a remarkable biogeography (Vecchioni et al. 2019).

At present, the genus *Tigriopus* includes 15 valid species with wide geographical distributions. They inhabit rock pools of Macquarie Island (*T. angulatus* Lang, 1933), Angola (*T. brachydactylus* Candeias, 1959), the North Atlantic Ocean (*T. brevicornis* Müller, 1776), the Pacific coast of North America (*T. californicus* Baker, 1912), Crozet Island (*T. crozettensis* Soyer, Thiriot-Quievreux & Colomines, 1987), the Mediterranean Sea (*T. fulvus* Fischer, 1860), Japan (Bonin Islands *T. igai* Itô, 1977); Shimoda, (*T. japonicus* Mori, 1938), Kerguelen Island (*T. kerguelensis* Soyer, Thiriot-Quievreux & Colomines, 1987), Antarctica (*T. kingsejongensis* Park, S. Lee, Cho, Yoon, Y. Lee & W. Lee, 2014), Senegal (*T. minutus* Bozic, 1960), New Zealand (*T. raki* Bradford, 1967), and Thailand (Rayong, *T. sirindhornae* Chullasorn, Dahms & Klangsin, 2013; Bangsaen, *T. thailandensis* Chullasorn, Ivanenko, Dahms, Kangtia & Yang, 2012).

Identification of Iranian species based on Wells (2007) and comparison with the description and illustrations of *T. raki* suggested that the Iranian specimens might be *T. raki*. However, the great distance between New Zealand and Iran and totally different ecological factors of these two areas made it doubtful the Iranian material was *T. raki*. Therefore, the paratype material of *T. raki* (NIWA [National Institute of Water and Atmospheric Research] 1610 P-33) was obtained and reinspected for a more precise identification of the Iranian specimens. Closer examination revealed important differences between *T. raki* and the Iranian material.

During an investigation on the intertidal copepod fauna of the Persian Gulf and the Oman Sea, a new member of the genus *Tigriopus* was discovered. Herein, we describe a new species of *Tigriopus*, *T. iranicus* sp. nov., which was found in rock pools in the Persian Gulf and the Oman Sea.

The description of some characters of *T. raki* were omitted in the original description, and a complementary redescription of the species is provided herein.

## Material and methods

The studied material was collected from rock pools in the Persian Gulf and the Oman Sea during a short-term research project in 2016 on the harpacticoid fauna of Iran. Collected specimens were preserved in 96% ethanol for future investigation. One male and one female were stained in a 1:1 solution of Congo Red and Acid Fuchsin for 24 h (Michels and Büntzow 2010). These materials were scanned using a Leica TCS SP5 equipped with a Leica DM5000 B upright microscope and 3 visible-light lasers (DPSS 10 mW 561 nm; HeNe 10 mW 633 nm; Ar 100 mW 458, 476,488, and 514 nm), in combination with the LAS AF 2.2.1 software (Leica Application Suite Advanced Florescence). Confocal Laser Scanning Microscopy images were obtained applying 561-nm excitation wave-length with 80% acousto-optic tunable filter. The acquisition resolution was 2048 × 2048. Final images, gained by maximum projections, were composed and adjusted for contrast and brightness using Adobe Photoshop CS6.

Whole male and female specimens were used for the illustration of the dorsal and lateral views of the habitus. The material was then dissected using a Leica MZ12 stereomicroscope for a detailed description of mouth parts and appendages. Dissected appendages were mounted on permanent slides with glycerin as mounting medium and sealed with a mixture of honeybee wax and paraffin. Pencil drawings of dissected parts were prepared with a Leica DMR differential interference contrast microscope equipped with a drawing tube at a magnification of 1000×. Digital inking was done using Adobe Illustrator CS6. The type material was deposited in the collection of the Senckenberg Gesellschaft für Naturforschung (Frankfurt/Main, Germany). The descriptive terminology follows Huys and Boxshall (1991) and Schminke (1976). Abbreviations used in the text: A1, antennule; A2, antenna; ae, aesthetasc; P1–P6, first to sixth swimming legs; enp, endopod; exp, exopod; enp-1, 2, 3, proximal, middle, distal segments of endopod; exp-1, 2, 3, proximal, middle, distal segments of exopod.

## Results

### Systematics

#### Order Harpacticoida Sars, 1903

**Family Harpacticidae Dana, 1846**

**Genus *Tigriopus* Norman, 1869**

##### Tigriopus iranicus sp. nov.

Taxon classificationAnimaliaHarpacticoidaHarpacticidae

56BB20C5-AA32-5583-8A5A-7E8A652B56F1

http://zoobank.org/D13668C8-8CE2-4594-829E-E67098C81F8C

Figs 1 , 2 , 3 , 4 , 5 , 6 , 7 , 8 , 9 , 10

###### Type material.

***Holotype*:** one adult female (SMF 37258/1-13) dissected, mounted on 13 slides. ***Allotype***: one male (SMF 37259/1-11) dissected, mounted on 11 slides, and 115 paratypes (65 females and 50 males) preserved in alcohol (SMF 37260).

###### Type locality.

Rock tidal pool on the coast of Iran, Jask, Vanak, 25°32'5"N, 58°52'12"E.

###### Differential diagnosis.

With marked distinction between prosome and urosome. P1-bearing somite fused to cephalothorax. Female antennule nine-segmented; seven-segmented and chirocerate in male. Mandible with two naked setae on basis, palp with two-segmented endopod and exopod. Maxilla with two endites, each with two setae. P1enp-3 with one claw, one spine, and one naked seta. P1exp-3 with five well-developed claws. Endopodal lobe of female P5 with four pinnate setae. Female P6 with two setae. Inner seta of the male P2enp-2 incorporated to the segment creating curved, pinnate process. Male P5 baseoendopods fused, forming a continuous plate, endopodal lobe without armature, exp with four elements.

###### Description.

**Female.** Total body length 630 µm, measured from tip of rostrum to posterior margin of furcal rami. Prosome four-segmented (Figs 1A–C, 2A, B), consisting of cephalothorax and three free pedigerous somites. First pedigerous somite bearing P1 fused to cephalosome. Cephalothorax and pedigerous somites smooth, furnished with sensilla; hyaline frills smooth without spinules or denticles. Rostrum (Fig. 3A) well developed, as long as first segment of antennule, bell-shaped, with two pairs of sensilla near apical and lateral margins. Urosome (Figs 1A–C, 2A, B) five-segmented, comprising fifth pedigerous somite, genital double-somite, two free abdominal somites and telson. Genital double-somite completely fused dorsally and ventrally, subdivided laterally by internal chitinous rib. Urosome ornamented with row of spinules laterally. Genital double-somite and first free abdominal somite clothed with sensilla dorsally. First and second free abdominal somites with posterior row of spinules ventrally. Telson with spinular rows ventrally and laterally, with two sensilla on dorsal distal margin. Anal operculum semicircular, smooth (Fig. 2A).

***Furcal rami*** (Figs 2A, B, 7A). Divergent, slightly longer than wide, furnished with oblique spinular row on dorsal surface and row of spinules ventrolaterally as illustrated. Seta I located on outer margin, approximately at mid-length of ramus; seta II positioned dorsally; seta III longer than seta II, placed on outer distal corner; seta IV pinnate; seta V longest and pinnate; seta VI as long as seta III, positioned on inner corner; seta VII tri-articulated, located dorsally.

***Antennule*** (Fig. 3B). Nine-segmented; first three segments longer than six apical segments combined; first segment with row of spinules; fourth segment with one seta fused basally to aesthetasc and two naked setae; apical acrothek on last segment with two setae fused at their bases and one aesthetasc. Armature formula: 1(1), 2(9), 3(7), 4(3+(1+ae)), 5(1), 6(4), 7(1), 8(3), 9(5+acrothek).

***Antenna*** (Fig. 3C). Three-segmented, composed of coxa, allobasis, and one free endopodal segment. Coxa without ornamentation. Allobasis ornamented with row of spinules and long setules proximally, abexopodal seta bipinnate. Free endopodal segment ornamented with row of spinules close to insertion site of proximal lateral spine, and with frill and minute spinules on outer distal corner; with two pinnate spines on lateral margin, one pinnate spine, four geniculate and two tiny naked setae distally. Exopod three-segmented; first segment longest with two pinnate setae; second segment with one pinnate seta; last segment with one pinnate seta incorporated into segment, and one tiny bare seta with spinules at its base.

***Labrum*** (Fig. 4A). Broad and well developed, triangular in lateral view, ornamented with setules along outer margin.

***Mandible*** (Fig. 4B). Coxa with well-developed gnathobase. The latter with six blunt and two multicuspidate teeth and one pinnate seta. Basis rectangular with two smooth setae. Both exopod and endopod two-segmented. Enp-1 with three naked setae laterally, two of them fused basally; enp-2 with five naked setae on distal margin. Exp-1 with two smooth setae on inner margin and spinules on outer margin; exp-2 with three naked setae.

***Maxillule*** (Fig. 4C, D). Arthrite of praecoxa with one ventral pinnate spine, tiny apical spine, two pinnate and four naked apical spines, and two smooth surface setae, ornamented with spinules on posterior surface, and with few lateral spinules medially. Coxal endite (Fig. 4D) cylindrical, with three naked setae, with spinules subdistally. Basis with two lateral smooth setae, and one pinnate and three smooth distal setae. Endopod one-segmented, cylindrical, with three naked setae. Exopod one-segmented, with four naked setae.

***Maxilla*** (Fig. 4E). Syncoxa bearing one praecoxal and one coxal endite, each armed with two setae, with two lobes (probably the remnant of endites) between syncoxal endites, with spinules on praecoxal endite, with some medial and some distal spinules on syncoxa. Allobasis prolonged into a strong pinnate claw, with one anterior naked seta and one posterior slender smooth, and one strong bipinnate seta. Endopod one-segmented, carrying two naked setae.

***Maxilliped*** (Fig. 3D). Syncoxa with spinule patch on outer margin and with distal spinule row; with one naked inner distal seta. Basis with strong spinules on inner margin and one naked seta. Endopod drawn out into a developed pinnate claw carrying a process with two smooth setae.

***Swimming legs P1–P4*** (Figs 5A, B, 6A, B) biramous, each ramus three-segmented and well-ornamented with spinules, exopod longer than endopod. Setal formula as follows:

**Table:** 

	Coxa	Basis	Exopod	Endopod
Leg 1	0–0	I–I	I–0; I–1; V	0–1, 0–0; II+1
Leg 2	0–0	I–0	I–1; I–1; III, II,2	0–1; 0–1; I,2,1
Leg 3	0–0	1–0	I–1; I–1; III, II,2	0–1; 0–1; I,2,1
Leg 4	0–0	1–0	I–1; I–1; III, II,3	0–1; 0–0; I,2,1

***P1*** (Fig. 5A). Intercoxal sclerite rectangular, longer than wide and smooth. Praecoxa and coxa ornamented with spinular rows on distolateral anterior surface. Basis bearing one inner and one outer spinulose flagellate spine, with rows of spinules on anterior surface as shown. Endopod approximately as long as first exopodal segment; enp-1 longest, with one long inner seta inserted at two-thirds length of segment, ornamented with setules and spinules as depicted, with spinule row on inner and outer margins; enp-2 wider than long, unarmed, with few large outer spinules; enp-3 with one strong curved pinnate claw, one spine and one tiny smooth seta at base of the claw, with outer spinular row. Exp-1 longest, bearing one pinnate flagellate outer spine distally, with row of outer spinules; Exp-2 ornamented with setules and spinules as shown, with one outer pinnate flagellate spine at two-thirds length of segment, and one inner subapical pinnate seta; exp-3 shortest, armed with five claw-like spines.

***P2*** (Fig. 5B). Intercoxal sclerite smooth, longer than wide and curved. Coxa triangular, with proximal and outer subdistal spinular rows, with distal frill on anterior surface, and with pore near inner margin. Basis with spinules at base of outer spine and at base of exopod, with inner distal frill and pore close to insertion site of exopod, with outer flagellate pinnate spine. Endopodal segments with strong outer spinules; enp-1 and -2 with one inner seta ornamented with setules proximally and with small spinules distally; enp-3 with one spinulose and two pinnate setae, and one blunt pinnate spine. Exopodal segments with strong outer spinules; exp-1 and exp-2 with anterior distal frill, with one outer naked spine and one inner pinnate seta; exp-3 with three naked outer spines, one outer distal element ornamented as shown and one inner distal pinnate seta, and two pinnate inner setae.

***P3*** (Fig. 6A). Coxa with spinules on outer distal corner and frill on inner distal border. Basis with strong outer spinules, with smooth outer seta. Segments of both rami with spinules on outer margins. Enp-1 and enp-2 armed with one long inner seta ornamented as depicted; enp-1 with, enp-2 without inner distal frill; enp-3 with three pinnate setae and one unipinnate spine. Exp-1 and exp-2 with anterior frill distally, with one outer pinnate spine and one inner seta ornamented as figured; exp-3 with three outer pinnate spines, one outer and one inner distal element as shown, and two inner setae.

***P4*** (Fig. 6B). Coxa with anterior frill distally and with outer spinules. Both exopodal and endopodal segments ornamented with outer spinules. Enp-1 with frill on anterior distal margin, bearing one inner pinnate seta; enp-2 unarmed; enp-3 with one pinnate spine and three pinnate setae. Exp-1 with distal frill and outer pore on anterior surface, with one pinnate outer spine and one pinnate inner seta; exp-2 with anterior distal frill, with pinnate outer spine and pinnate inner seta; exp-3 with three outer pinnate spines, one outer and one inner distal element, and three inner setae of which medial spinulose.

***P5*** (Fig. 7B). Exopod and baseoendopod separate; ornamented with spinules as illustrated; baseoendopod bearing smooth outer basal seta; endopodal lobe with four pinnate setae, with one tube-pore near innermost seta and two pores on anterior surface; exopod rectangular, with five pinnate setae and one pore on anterior surface.

***P6*** (Fig. 7A). One-segmented, bearing one naked and one pinnate seta.

***Genital field*** (Fig. 7A). Situated in the middle of the genital double-somite, with one median genital pore at the boundary between genital somite and first abdominal somite.

**Male** (Fig. 8A–C). Habitus as in female except for genital somite separated from first abdominal somite. Total body length 685 µm measured from tip of rostrum to posterior margin of furcal rami. Sexual dimorphism expressed in antennule, antenna (the latter as in female, but without abexopodal seta), P2, P5 and P6.

***Antennule*** (Fig. 9A, B). Seven-segmented, chirocerate, with geniculation between sixth and seventh segments. First segment with anterior spinules; segment six largest and swollen, with four multicuspidate elements ventrally; segment seven with acrothek and several denticles on anterior surface. Armature formula: 1(1), 2(1), 3(11), 4(1), 5(1), 6(11+(1+ae)), 7(7+acrothek).

***P2*** (Fig. 10A). Praecoxa small and triangular. Coxa and basis as in female, except for basis without anterior pore. Exopod as in female except for additional anterior pore on exp-1. Endopod three-segmented; enp-1 largely as in female; enp-2 with outer apophysis, inner element comparatively shorter than in female and robust, incorporated into the segment, with row of inner strong spinules; enp-3 small, with three pinnate and one small naked seta.

***P5*** (Fig. 10B). Baseoendopods of both legs fused forming continuous plate; endopodal lobe completely incorporated into segment, unarmed, with medial transverse spinular row as shown; outer basal seta naked, with spinules at its base. Exopod rectangular, ornamented with anterior and posterior spinules, with four elements, innermost longest.

***P6*** (Fig. 10C). Symmetrical, represented by two pinnate and one naked seta, ornamented with spinules.

###### Etymology.

The specific epithet *iranicus* refers to the country where the new species was found. It is in the nominative singular. Gender masculine.

##### Tigriopus raki

Taxon classificationAnimaliaHarpacticoidaHarpacticidae

Bradford, 1967

3FD7F342-C33E-54C6-94BE-E139264D30E6

###### Material examined.

One female (SMF 37261/1-13) and one male (SMF 37262/1-10) paratype (NIWA 1610 P-33) dissected and mounted on slides, and three females and two males preserved in ethanol (SMF 37263).

###### Type locality.

Leigh, north of Auckland, at about 36°30'S, 174°45'E on the east coast. Habitat: marine, in splash zone pool.

###### Redescription.

**Female.** Total body length 560 µm, measured from tip of rostrum to posterior margin of furcal rami. Few sensilla scattered on body surface (Fig. 12A, B).

**Figure 11.**
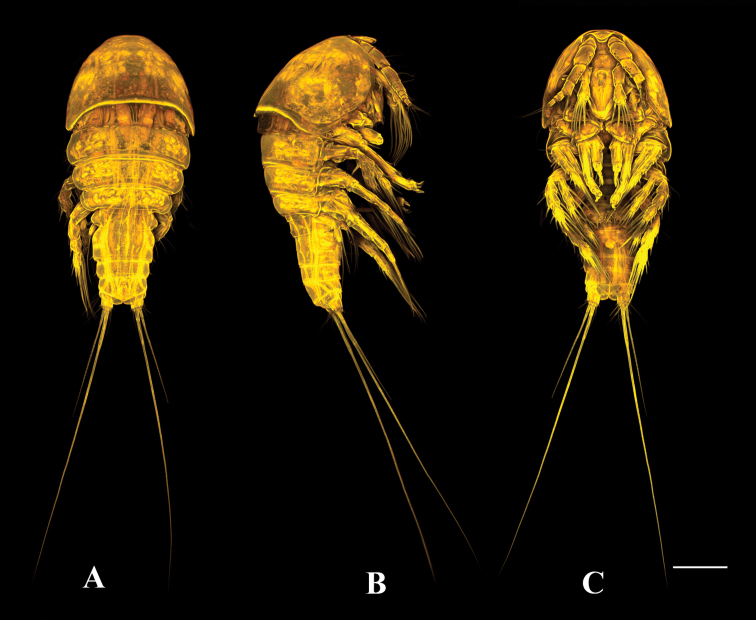
*Tigriopus raki*. female. Confocal laser microphotograph **A** habitus, dorsal **B** habitus, lateral **C** habitus, ventral. Scale bar: 100 µm.

**Figure 12.**
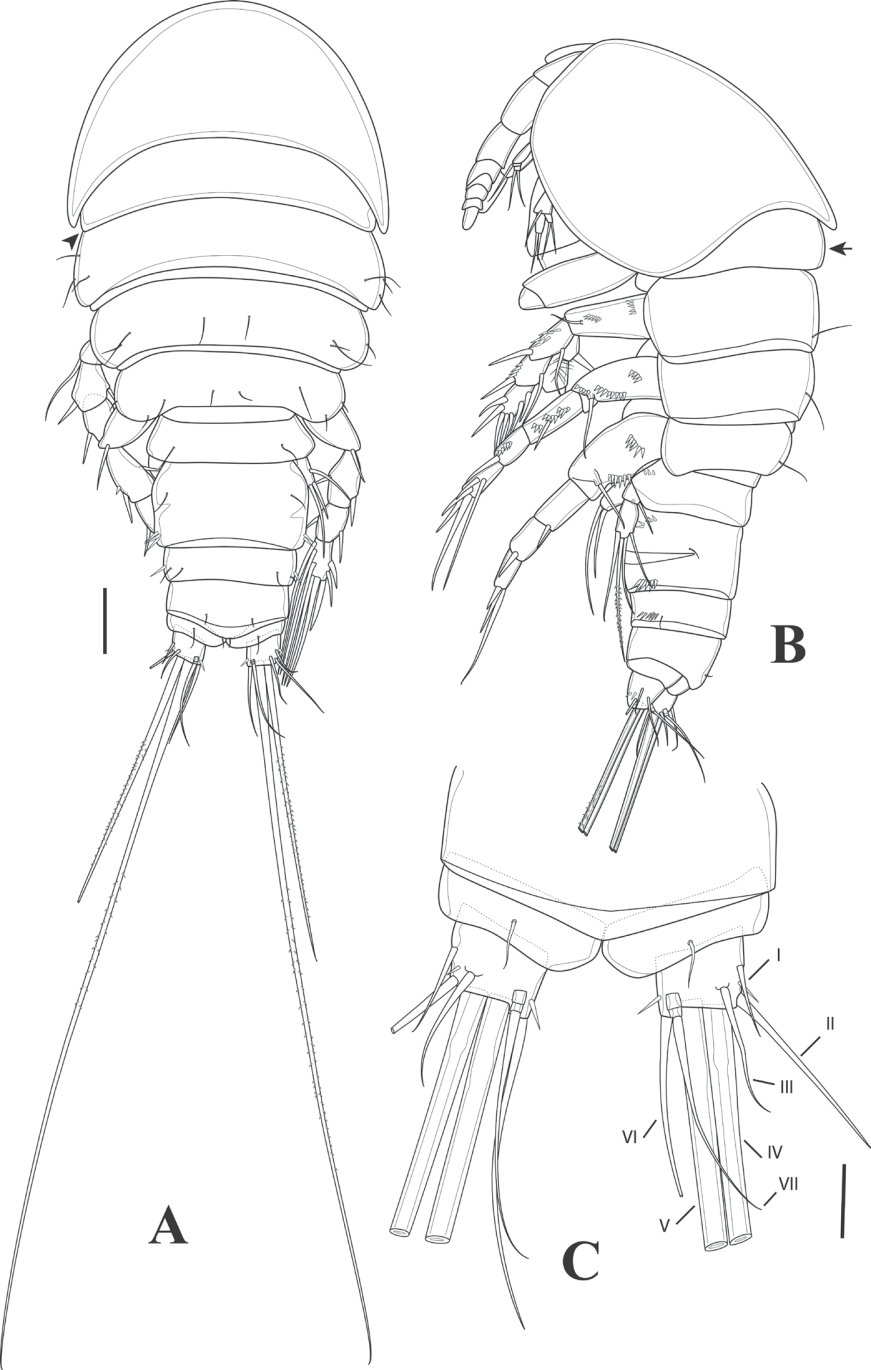
*Tigriopus raki* female **A** habitus, dorsal (tergite of first pedigerous somite arrowed) **B** habitus, lateral (tergite of first pedigerous somite arrowed) **C** anal somite and furcal rami, dorsal. Scale bars: 50 µm (**A, B**), 20 µm (**C**).

**Figure 13.**
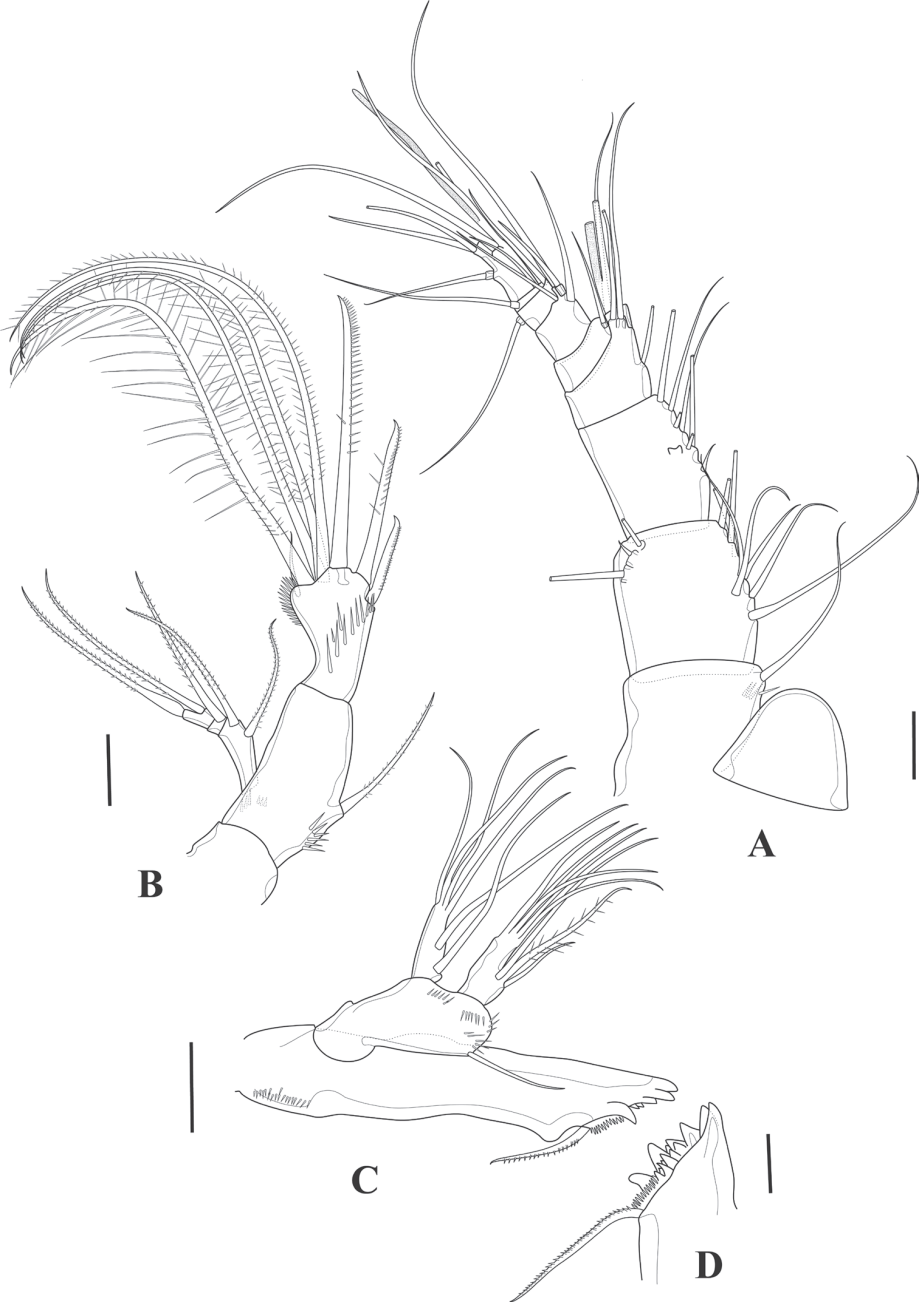
*Tigriopus raki* female **A** rostrum and antennule **B** antenna **C** mandible **D** cutting edge of mandible. Scale bars: 20 µm (**A–C**), 10 µm (**D**).

***Prosome*** (Figs 11A, B, 12A, B). Four-segmented, including cephalothorax with first pedigerous somite incorporated, and three free pedigerous somites. Tergite of first pedigerous somite remarkable from dorsal and lateral view (Figs 11A, B, 12A, B; marked by an arrow in Fig. 12A, B). Hyaline frills on posterior and lateral margins smooth. ***Rostrum*** (Fig. 13A) bell-shaped, defined at base, without sensilla.

***Urosome*** (Figs 11A, C, 12A, B). Five-segmented, comprising fifth pedigerous somite, genital double-somite, two free abdominal somites and telson. Genital double-somite completely fused dorsally and ventrally, boundary between two somites slightly distinguishable by lateral internal chitinous rib. With spinulose rows on first and second abdominal somites. Anal operculum semicircular, smooth.

***Furcal rami*** (Fig. 12C) slightly wider than long; with outer and distal spinulose rows. Lateral seta I smooth, implanted in middle of ramus. Seta II smooth, longer than seta I, located subdistally. Seta III smooth, displaced ventrally, subdistal. Setae IV and V pinnate, located on distal margin. Seta VI smooth inserted on distal, inner corner. Seta VII tri-articulated and smooth, dorsally located near distal inner margin.

***Antennule*** (Fig. 13A). Nine-segmented. All segments smooth except for first segment with few spinules. Five apical segments shorter than first two segments combined. Segment eight very small. Ninth segment with apical acrothek. Armature formula: 1 (1), 2(12), 3(8), 4(4+(1+ae)), 5(1), 6(4), 7(3), 8(1), 9(5+acrothek).

***Antenna*** (Fig. 13B). Consisting of coxa, allobasis and one free endopodal segment. Coxa without ornamentation. Allobasis longest, with proximal inner spinules, with pinnate abexopodal seta. Exopod three-segmented; first segment longest with two pinnate setae; second segment shortest, with one pinnate seta; last segment bearing two pinnate setae. Free endopodal segment ornamented with long spinules and with outer distal frill, with two lateral and one subdistal inner spine, four apical pinnate setae, and one tiny smooth outer distal seta.

***Mandible*** (Fig. 13C, D). Coxa with anterior row of spinules. Gnathobase well developed; with two rows of blunt teeth and a comb-like structure; with one long pinnate seta. Basis ornamented with spinules; with one naked seta. Both rami one-segmented. Endopod with two pinnate and one naked seta laterally; with five distal setae fused at their bases. Exopod with two lateral setae, and four apical naked elements fused basally to segment.

**Figure 14.**
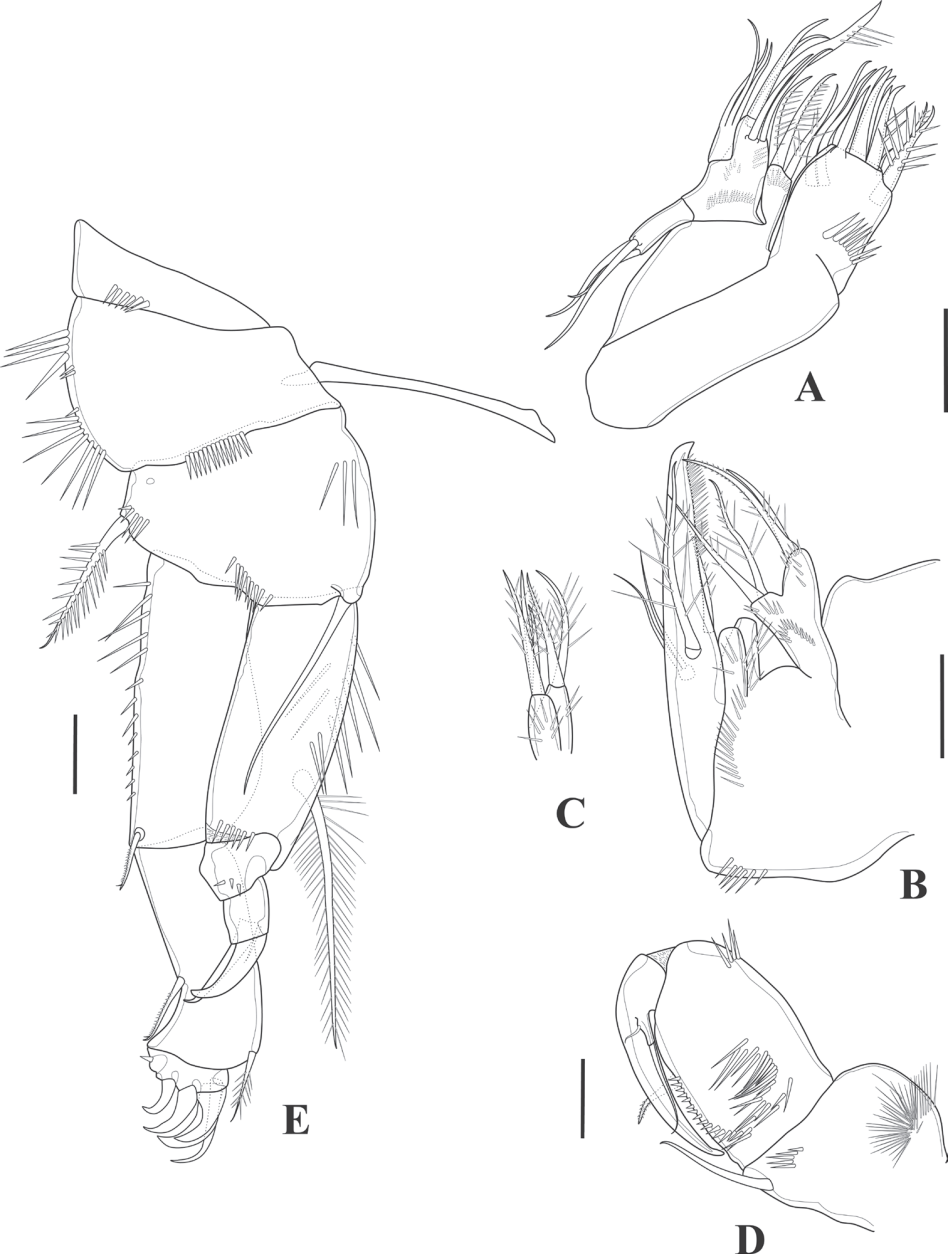
*Tigriopus raki* female **A** maxillule **B** maxilla **C** medial and distal syncoxal endites of the maxilla **D** maxilliped **E**P1. Scale bars: 20 µm.

***Maxillule*** (Fig. 14A). Arthrite of praecoxa well developed; ornamented with spinules; bearing one tiny and eight developed spines apically, with two surface setae. Coxal endite with spinular row on distal margin; with three spinulose setae. Basis ornamented with spinules on anterior and posterior surface; with three subdistal naked setae, and two distal elements of which one visibly stronger and spinulose. Endopod with three naked setae fused to segment. Exopod rectangular, armed with three naked setae.

***Maxilla*** (Fig. 14B, C). Syncoxa with three endites; ornamented with subdistal medial and outer spinules; praecoxal endite bilobed, each lobe with two pinnate setae; coxal endites (Fig. 14C) with two and three pinnate setae, respectively. Allobasis with strong unipinnate claw and one posterior and one anterior strong pinnate seta. Endopod one-segmented, with two naked setae.

***Maxilliped*** (Fig. 14D). Syncoxa ornamented with hair-like outer spinules and with inner spinules; with one naked inner seta. Basis with medial spinules of variable lengths; with one spinulose seta. Endopod drawn out into strong claw; with one cylindrical process carrying one naked seta apically and one tiny naked seta basally.

**Figure 15.**
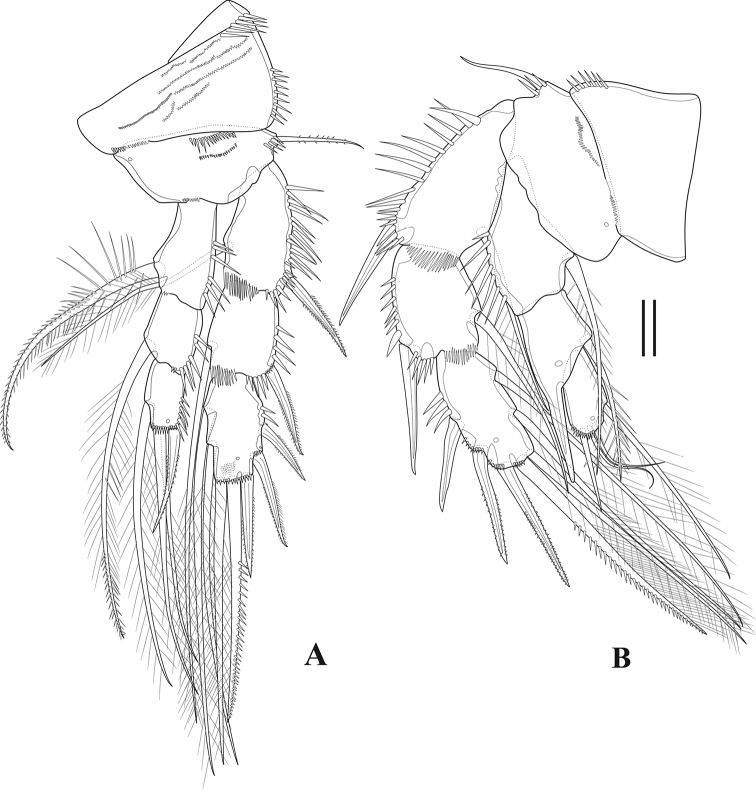
*Tigriopus raki* female **A**P2**B** male. P2. Scale bar: 20 µm.

**Figure 16.**
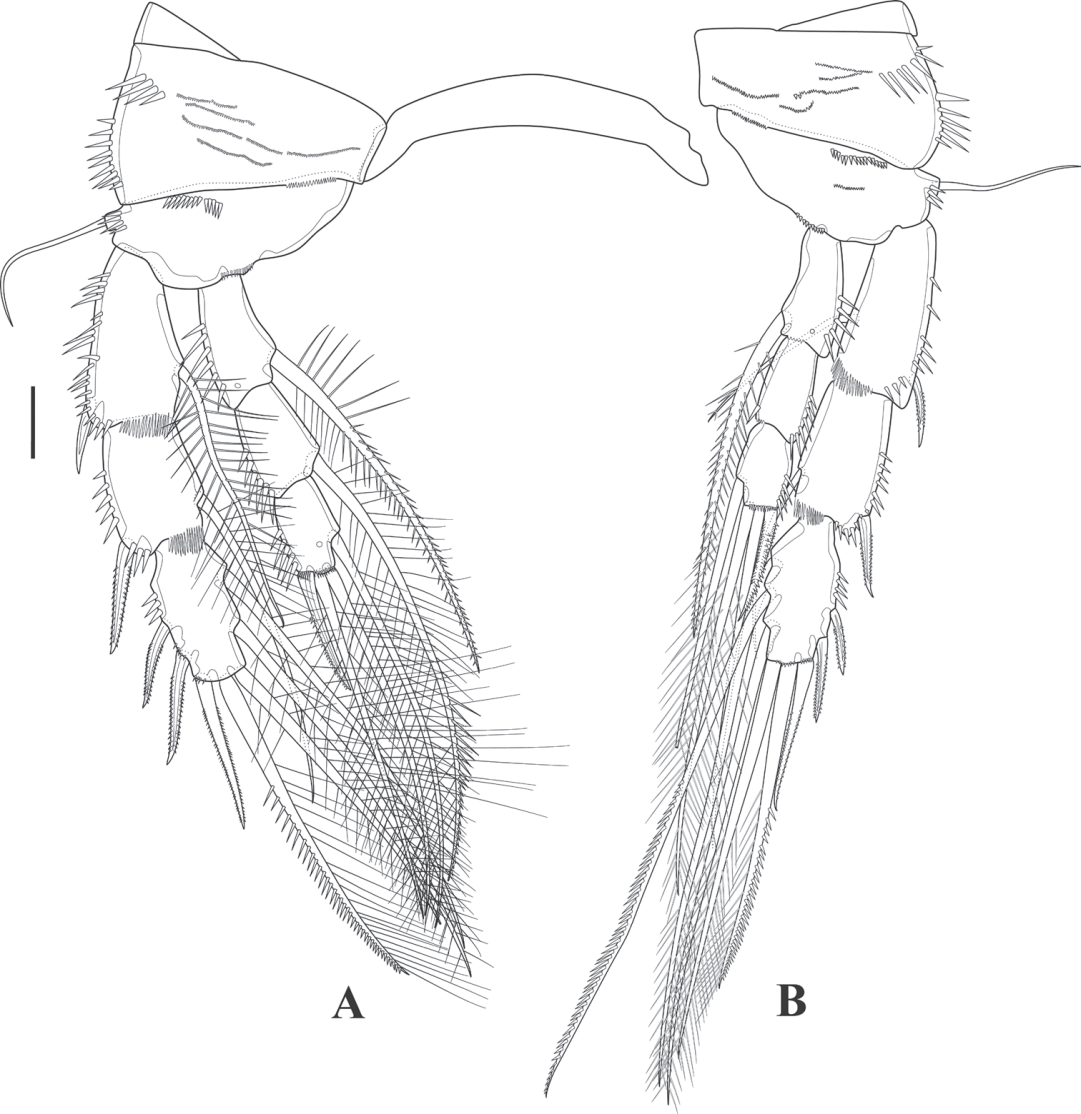
*Tigriopus raki* female **A**P3**B**P4. Scale bar: 20 µm.

***Swimming legs 1–4*** (Figs 14E, 15A, 16A, B) biramous; rami three-segmented; ornamented with spinules of different sizes; exopod slightly longer than endopod. Setal formula as follows:

**Table:** 

	**Coxa**	**Basis**	**Exopod**	**Endopod**
Leg 1	0–0	1–1	I–0; I–1; V	0–1, 0–0; II+1
Leg 2	0–0	1–0	I–1; I–1; III, II, 2	0–1; 0–1; I,2,1
Leg 3	0–0	1–0	I–1; I–1; III, II, 2	0–1; 0–1; I,2,1
Leg 4	0–0	1–0	I–1; I–1; III, II, 3	0–1; 0–0; I,2,1

***P1*** (Fig. 14E). Intercoxal sclerite slender and smooth. Praecoxa triangular, with anterior medial spinules. Coxa ornamented with large outer and distal medial spinules. Basis with spinules on anterior surface; outer seta stout and pinnate; inner seta long and naked. Endopod shorter than first two exopodal segments combined; enp-1 approximately as long as exp-1, with one inner long pinnate seta at two-thirds length of segment, with long inner spinules; enp-2 and enp-3 small, enp-2 unarmed, enp-3 armed with two strong claws and one tiny seta at base of claw. Exp-1 with spinules along outer margin, outer flagellate spine unipinnate; exp-2 with curved outer margin, with one pinnate outer spine at about mid-length, and one pinnate inner distal seta; exp-3 reduced, with four claws and one spine.

***P2*** (Fig. 15A). Praecoxa small and triangular, with spinules as shown. Coxa ornamented with spinules. Basis with pinnate outer seta and anterior spinules. Endopodal segments with spinules on outer margin; enp-1 and enp-2 with long pinnate inner seta; enp-3 with three pinnate setae and one pinnate spine. Exp-1 and -2 armed with one outer pinnate spine and one pinnate inner seta; exp-3 with three outer pinnate spines, one outer and one inner element ornamented as shown, and two inner pinnate setae.

***P3*** (Fig. 16A). Intercoxal sclerite rectangular, smooth and longer than wide. Similar to P2, except praecoxa unornamented and basal outer seta naked.

***P4*** (16B). Praecoxa triangular and unornamented. Coxa and basis largely as in P3. Enp-1 with one large pinnate inner seta; enp-2 unarmed; enp-3 with three pinnate setae and one pinnate spine. Exp-1 and -2 each with one outer pinnate spine and one inner pinnate seta; exp-3 with three pinnate outer spines, one outer and inner distal element ornamented as shown, and three pinnate setae.

**Figure 17.**
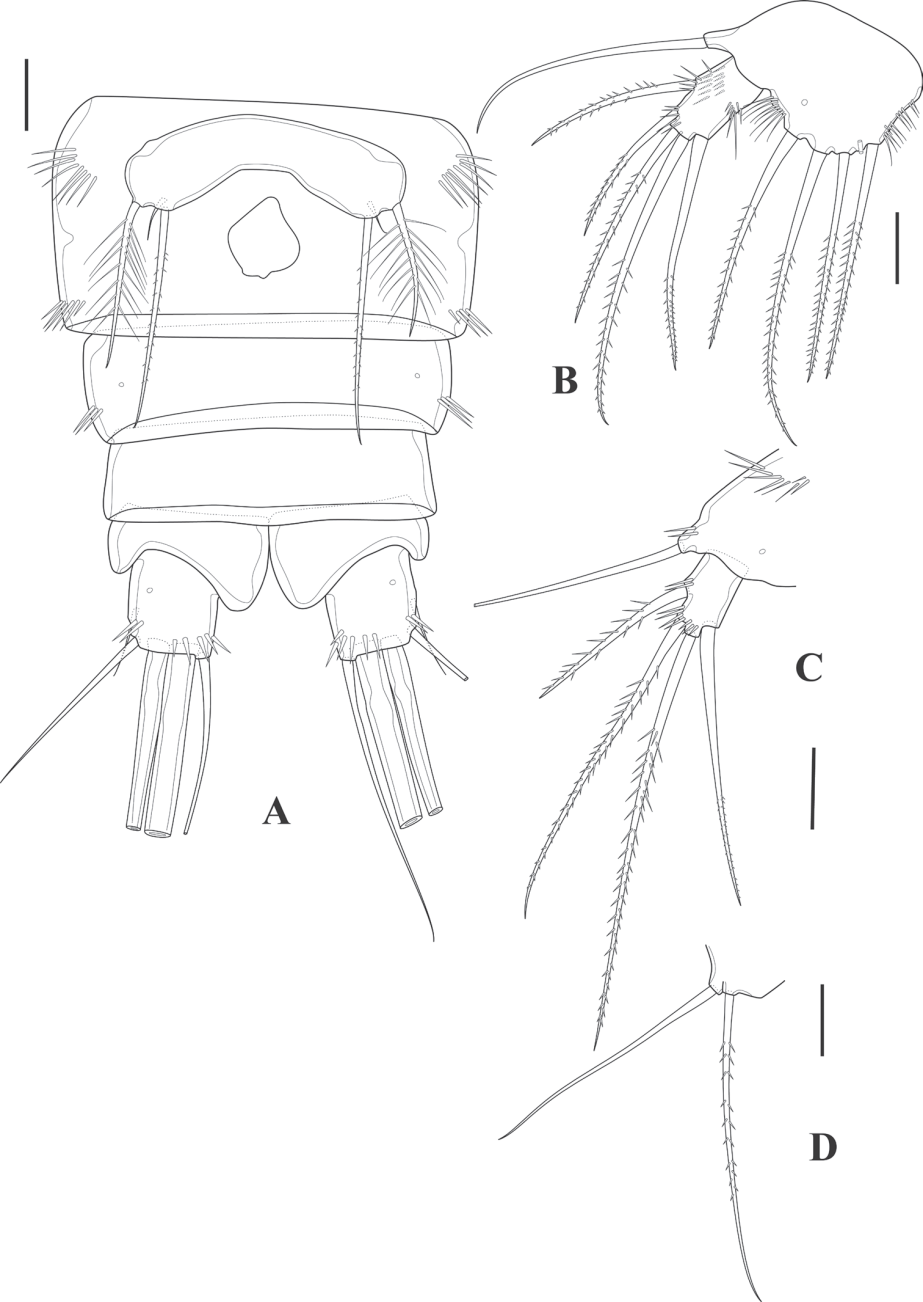
*Tigriopus raki* female **A** urosome, ventral **B**P5. Male **C**P5**D**P6. Scale bars: 20 µm.

***P5*** (Fig. 17B). Baseoendopod and exopod separated. Basal outer seta long and naked. Endopodal lobe ornamented with long spinules along inner and outer margin, with inner tube-pore and median pore on anterior surface, with four pinnate setae. Exopod rectangular, longer than endopodal lobe, furnished with spinules on anterior and posterior surface, with five pinnate setae.

***P6*** (Fig. 17A). Situated on anterior part of genital-double somite, with two pinnate and one small naked seta.

***Genital field*** (Fig. 17A). Copulatory pore approximately in middle of genital double-somite.

**Figure 18.**
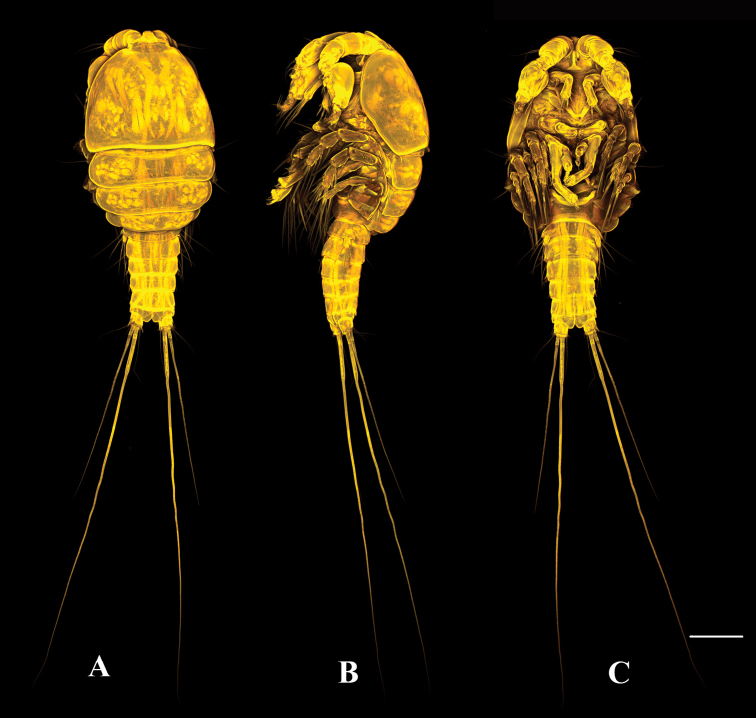
*Tigriopus raki* male. Confocal laser microphotograph **A** habitus, dorsal **B** habitus, lateral **C** habitus, ventral. Scale bar: 100 µm.

**Male** (Fig. 18A–C). Habitus as in female, except genital somite separated from first abdominal somite. Total body length 600 µm measured from tip of rostrum to posterior margin of furcal rami. Sexual dimorphism expressed in antennule, antenna (without abexopodal seta), P2, P5 and P6.

**Figure 19.**
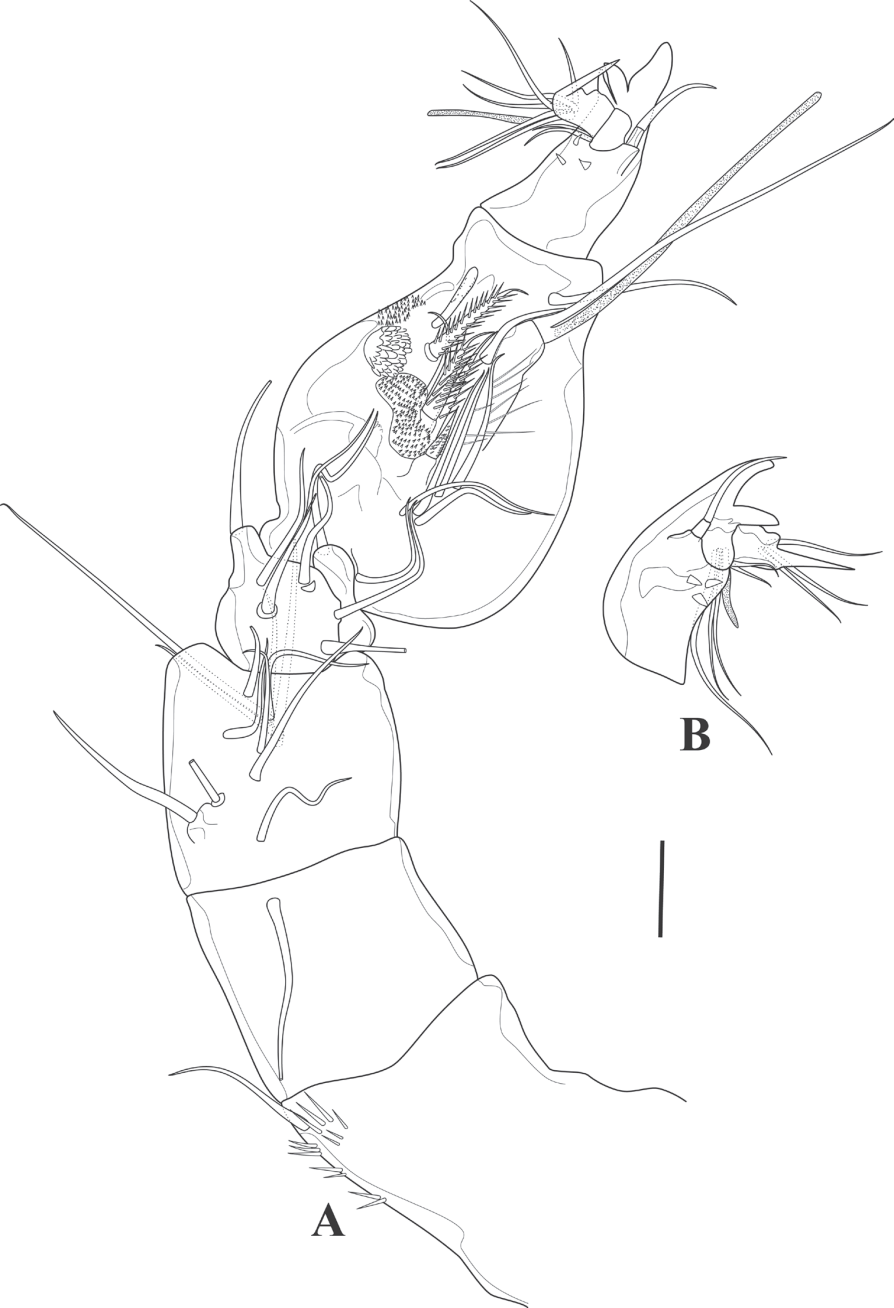
*Tigriopus raki* male **A** antennule, ventral **B** last segment of the antennule. Scale bar: 20 µm.

***Antennule*** (Fig. 19A, B). Seven-segmented, chirocerate; with geniculation between segments 6 and 7. First segment ornamented with spinules around seta. Segment 5 smallest. Segment 6 largest and swollen, with three multicuspidate elements and one club-like element ventrally. Segment 7 with membranous element and three ventral teeth. Armature formula as follows: 1 (1), 2(1), 3(12), 4(7), 5(1), 6(13+(1+ae)),7(7+acrothek).

***P2*** (Fig. 15B). Coxa with spinules and frill anteriorly. Basis with anterior frill, and with outer naked seta. Enp-2 with outer apophysis and pinnate inner seta. Enp-3 with one pinnate inner seta, two naked reduced apical setae, and one naked spine.

***P5*** (Fig. 17C). Baseoendopod of both legs fused, endopodal lobe completely absorbed into segment, unarmed. Basal seta naked and long. Exopod rectangular with four strong pinnate setae.

***P6*** (Fig. 17D). Symmetrical, with one outer naked and one inner pinnate seta.

### Diagnostic key to the species of *Tigriopus*

**Table:** 

1	Male antenna with abexopodal seta	**2**
–	Male antenna without abexopodal seta	**3**
2	Male P2enp-2 with well-developed knob	***T. kingsejongensis***
–	Male P2enp-2 without knob	***T. kerguelensis***
3	Female P5 enopod with five setae	**4**
–	Female P5 enopod with four setae	**11**
4	Male P5 exopod with five setae	**5**
–	Male P5 exopod with four setae	**7**
5	P4exp-3 with seven setae/spines	**6**
–	P4exp-3 with eight setae/spines	**10**
6	P1exp-3 with four claws and one pinnate seta	***T. japonicus***
–	P1exp-3 with five claws	***T. californicus***
7	Mandibular basis with one seta; female with two copulatory apertures	**8**
–	Mandibular basis with two setae; female with one copulatory aperture	**9**
8	Male antennule eight-segmented; coxal endite of the maxillula with three setae; endopod of the maxilla with four setae	***T. thailandensis***
–	Male antennule seven-segmented; coxal endite of the maxillula with five setae; endopod of the maxilla with two setae	***T. sirindhornae***
9	Mandibular endopod with eight setae	***T. crozettensis***
–	Mandibular endopod with seven setae	***T. angulatus***
10	P1enp-3 with one claw and two setae	***T. brevicornis***
–	P1enp-3 with two claws	***T. fulvus***
11	Maxillary syncoxa with three endites	**12**
–	Maxillary syncoxa with two endites	***T. iranicus* sp. nov.**
12	Antennary exp-1 with two setae	**13**
–	Antennary exp-1 with one seta	***T. brachydactylus***
13	Male P5 baseoendopod without seta	**14**
–	Male P5 baseoendopod with one seta	***T. minutus***
14	Maxillary endopod with four setae; mandibular basis with two setae	***T. igai***
–	Maxillary endopod with three setae; mandibular basis with one seta	***T. raki***

## Discussion

The new species, *T. iranicus* sp. nov., was allocated into *Tigriopus* on account of the combination of: 1) a nine-segmented antennule in the female; 2) a three-segmented antennary exopod (with setal formula 2.1.2); 3) a three-segmented P1 endopod; 4) male P2enp-2 with outer apophysis; 5) P3 without sexual dimorphism, and 7) male P5 endopodal lobe reduced or absent.

*Tigriopus iranicus* sp. nov. is the third species of the genus reported from the Indian Ocean. *Tigriopus crozettensis* and *T. kerguelensis* were reported by Soyer et al. (1987) from Crozet and Kerguelen islands, respectively, in the southern Indian Ocean. The new species is the first record of *Tigriopus* from the northern part of the Indian Ocean. The species was found in tide pools in the Persian Gulf and the Oman Sea. Rocky shores are restricted to three areas of the Iranian southern coast (Polgar 2017).

The great similarities between females of different species make subtle details necessary for separation of species (Wells 2007). The new species is defined by the following autapomorphies: i) the number of syncoxal endites of the maxilla is reduced from three to two (the genus *Tigriopus* has been diagnosed with three endites, the proximal one of which is bilobed; *T. iranicus* sp. nov. is unique within the Harpacticidae in having a reduced number of syncoxal endites of the maxilla); ii) the reduced number from three to two (as in other species of the genus) setae on the female P6.

In addition to differences with other congeners, *T. iranicus* sp. nov., displays a unique two-segmented mandibular endopod. Within the genus *Tigriopus* the mandibular endopod is one-segmented. The two-segmented condition in the new species indicates a plesiomorphic state in the genus. In comparison with other species, the P1enp-3 armature of *T. iranicus* sp. nov. has a different armature. The P1enp-3 has one developed pinnate claw, one strong geniculate spine, and one slender seta, which are undescribed and not shown in any other species of the genus.

The new species, *T. brevicornis* and *T. minutus* (see Bozic 1960: 195, fig. 9) share the incorporation of the inner element of the male P2enp-2 into the segment to form a curved pinnate spinous process. Similarly, the male P5 of the new species, *T. raki* and *T. igai* share the complete absorption of the unarmed endopodal lobe and a tetrasetose exopod.

As most older descriptions of species are incomplete, it is difficult to make further comparisons. Nevertheless, based on available information, the new species is most closely related to *T. igai* and *T. raki*, and seems to be more closely related to the latter. In addition to the apomorphies of *T. iranicus* sp. nov., there are several significant differences that separate the new species from the other two species. The new species differs from *T. igai* in: i) the number of setae on the female P6; ii) the number of endopodal and exopodal segments of the mandible; iii) the number of setae on the exopod of the mandible; iv) the number of setae on the coxal endite of the maxillule; and v) the number of setae on the syncoxal endites of the maxilla. *Tigriopus iranicus* sp. nov. differs from *T. raki* in the presence of: i) two setae on the mandibular basis (one in *T. raki*); ii) six setae on the basis of the maxillule (five in *T. raki*); ii) four setae on the exopod of the maxillule (three in *T. raki*); iv) two setae on the proximal syncoxal endite of the maxilla (four in *T. raki*); and v) three setae on the allobasis of the maxilla (two in *T. raki*).

*Tigriopus raki* was originally described from splash-zone pools from Northland, New Zealand by Bradford (1967). Our redescription of *T. raki* upon careful examination of the paratype material allows the following amendments to Bradford’s (1967) original description of the species:

1. Bradford (1967) omitted the description and illustration of the maxillule and the maxilla. These appendages have been fully described and illustrated here.
2. Bradford (1967: 54, fig. 2h) observed only one seta accompanying the endopodal claw of the maxilliped; we observed two accompanying setae on the endopodal claw of maxilliped.
3. We observed that the male P2 enp-2 inner seta is not incorporated into the segment.

The number of species currently attributed to *Tigriopus* increases to 15. Following Soyer et al. (1987), *T. iranicus* sp. nov. belongs to a group of tropical species composed of *T. brachydactylus*, *T. igai*, *T. minutus*, and *T. raki*. A preliminary phylogenetic analysis (Nazari and Gómez unpubl.) supports such grouping.

## Supplementary Material

XML Treatment for Tigriopus iranicus

XML Treatment for Tigriopus raki

## Supplementary materials

Supplementary material 1 Geographic coordinates Data type occurrence https://binary.pensoft.net/file/536445This dataset is made available under the Open Database License (http://opendatacommons.org/licenses/odbl/1.0/). The Open Database License (ODbL) is a license agreement intended to allow users to freely share, modify, and use this Dataset while maintaining this same freedom for others, provided that the original source and author(s) are credited.Fatemeh Nazari, Omid Mirshamsi, Pedro Martínez Arbizu
